# Exotic Tourist Destinations and Transmission of Infections by Swimming Pools and Hot Springs—A Literature Review

**DOI:** 10.3390/ijerph15122730

**Published:** 2018-12-03

**Authors:** Athena Mavridou, Olga Pappa, Olga Papatzitze, Chrysa Dioli, Anastasia Maria Kefala, Panagiotis Drossos, Apostolos Beloukas

**Affiliations:** 1 Department of Biomedical Sciences, University of West Attica, 12243 Egaleo, Greece; olpap79@gmail.com (O.P.); olgapapat@hotmail.com (O.P.); chrysrose57@gmail.com (C.D.); anastasia.m.kefala@gmail.com (A.M.K.); pdrossos2006@yahoo.gr (P.D.); abeloukas@uniwa.gr (A.B.); 2 Central Public Health Laboratory, Hellenic Centre of Disease Control and Prevention, 15123 Maroussi, Greece; 3 West Attica General Hospital, “Santa Barbara”, 12351 Santa Barbara, Greece; 4 Institute of Infection and Global Health, University of Liverpool, Liverpool L69 3BX, UK

**Keywords:** swimming pools, tropics, subtropics, pool assessment, infectious diseases

## Abstract

A growing number of people undertake international travel, and yet faster growth of such travel is expected in the tropics. Information on the hazards presented by pool and hot spring waters in tropical countries is very limited. This review aims to collate available information on pool water quality, alongside data on cases and outbreaks associated with swimming in pools in tropical regions affecting both local populations and travellers. Bacteria species commonly causing cases and outbreaks in the tropics as well as elsewhere in the world were excluded, and the review focuses on studies related to pathogens that, with the exception of *Cryptosporidium*, are unusual in more temperate climates. Studies concerning subtropical countries were included in the light of climate change. Diseases transmitted by vectors breeding in poorly maintained, neglected or abandoned pools were also included. 83 studies dealing with Microsporidia, *Leptospira* spp., *Schistosomas* spp., *Cryptosporidium* spp., *Acanthamoeba* spp., *Naegleria* spp., *Clostridium trachomatis*, viruses, and vectors breeding in swimming pool and hot tub waters, and fulfilling predefined criteria, have been included in our survey of the literature. In conclusion, prevention strategies for pool safety in the tropics are imperative. Public health authorities need to provide guidance to westerners travelling to exotic destinations on how to protect their health in swimming pools.

## 1. Introduction

An increasing number of people undertake international travel for professional, social, recreational and humanitarian purposes. Nowadays, more people travel greater distances and at greater speed than ever before, and this upward trend looks set to continue. Internationally, tourist arrivals have increased from 25 million globally in 1950 to 1235 million in 2016 [1] with travel for leisure and pleasure accounting for more than half of international tourism arrivals [2].

According to the research project “Tourism Towards 2030”, the number of international tourist arrivals worldwide will increase by an average of 3.3% per year through to 2030. Greater growth is expected to occur in the tropical Asian and the Pacific regions, where arrivals are forecast to reach 535 million in 2030 (+4.9% per year). Countries of the tropical zone, such as those located in the Middle East and Africa regions are expected to double their arrival numbers during the same period, from 61 and 50 million to 149 and 134 million, respectively [1]. According to World Tourism highlights, 2001 the fastest developing region continues to be East Asia and the Pacific [3].

From a public health perspective, travellers are exposed to a variety of health risks in unfamiliar environments [2]. International travel can pose severe risks to health, depending both on travellers’ health needs and on the type of travel undertaken. Accidents continue to be the primary cause of morbidity and mortality for international travellers, but infections also present an important health risk. Moreover, travellers interact dynamically with microbes and places. Travellers can carry these microbes and their genetic material, and, as Baker stated,” can play multiple roles with regard to microbes, as victims, sentinels, couriers, processors, and transmitters of microbial pathogens” [4].

There is abundant information and guidance to travellers regarding precautions that need to be taken in respect of food, drinking water and air quality in tropical destinations. Nevertheless, information on the hazards presented by recreational and especially pool, spa and hot spring waters as a mode of transmission of pathogens is very limited, even though numerous infectious agents may threaten the health or comfort of pool and hot tub users [5]. As examples, the important World Health Organization (WHO) document [2] attributes only half, out of 244, pages to precautions related to the use of recreational waters [2]; the European Network on Imported Infectious Diseases Surveillance (TravelHealthPro) does not mention recreational waters on their webpage, which provides guidance to travellers on how to take care of their health [6]; the announced revision of the WHO Guidelines for recreational waters [3] has been suspended [3]; Page et al. do not refer to pool water in their outstanding review regarding attitudes of tourists towards water use in the developing world [7].

Climate changes are creating conditions in the subtropical zones similar to the tropics and these geographical regions were included in the review. For the purposes of our review, we considered both primary transmission from pool waters and secondary infections spread by the pool users. Tropical diseases encompass all diseases that occur solely, or principally, in the tropics. In practice, the term is often taken to refer to infectious diseases that thrive in hot, humid conditions, such as malaria, leishmaniasis, schistosomiasis, onchocerciasis, lymphatic filariasis, Chagas disease, African trypanosomiasis, and dengue [8]. Besides the “big three” diseases—malaria, tuberculosis, HIV/AIDS—which are well known causes of major global mortality, morbidity and burden, the term “neglected tropical diseases” has been introduced in the literature. They comprise a new field for travellers’ health and the list includes 40 helminth, bacterial, protozoan, fungal, viral and ectoparasitic infections affecting local populations in the tropics, which are strongly associated with poverty and socio-ecological systems, but also presenting a serious health risk for travellers [9]. It is worth noting, however, that many of the so-called “tropical” diseases are not transmitted through recreational waters.

### Objective

This review aims at collating information on pool water quality, and cases and outbreaks related to swimming in pools and hot springs in tropical and subtropical regions, and at carrying out a search and review of papers dealing with hazards deriving from the use of pools in the tropical and subtropical zones.

## 2. Materials and Methods 

### Search Strategy/Inclusion Criteria

Cochrane instructions for a systematic search that seeks to identify all studies dealing with incidences originating in tropical and subtropical countries were followed (https://ph.cochrane.org/ sites/ph.cochrane.org/files/public/uploads/HPPH_systematic_review_handbook.pdf). The studies were required to meet pre-defined eligibility criteria, a major one being transmission via pool, spa or hot spring waters or the detection of the pathogens in such waters. Thus, studies reporting pathogens without confirmed transmission through recreational waters were excluded. For instance, the *Chikungunya* virus was the aetiological agent of an outbreak in Kenya in 2004, and major outbreaks followed in Indian Ocean island countries such as Reunion, Mauritius, Comoros, Seychelles and Madagascar in 2005 to 2006 [4,10]. *Burkholderia pseudomallei* has also caused melioidosis in the tropics [11]. Nevertheless, so far neither of these two pathogens’ transmission has been confirmed to involve pool waters. 

Diseases transmitted by vectors breeding in poorly maintained, neglected or abandoned pools were also included. As tourism presents seasonality a high number of pools stay inactive for several months, often still containing the water of the past season. This situation encourages the proliferation of pathogens and the extensive use of these waters by vectors in order to breed. Thousands of flooded swimming pools were abandoned in New Orleans following Hurricane Katrina and provided a natural experiment to examine colonization of a novel aquatic habitat by mosquito larvae and their aquatic predators [12,13,14,15].

A large number of cases and outbreaks described in the literature surely derive from unidentified sources, among which a number is likely to have been from swimming pools: reports of this kind or with a preconceived bias regarding the mode of transmission were excluded from this review. 

**Figure 1:**
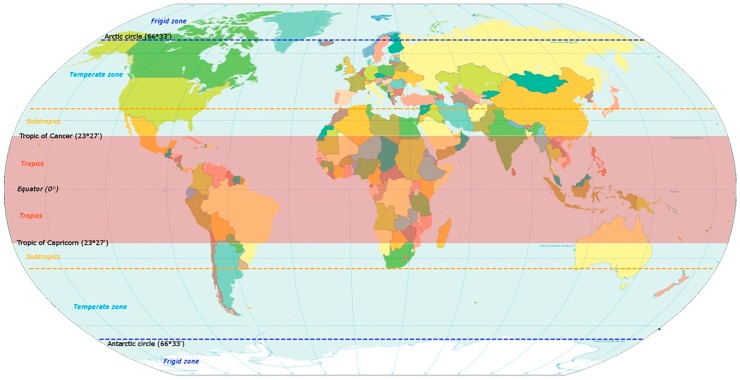
Map of the world indicating the tropical and subtropical zones.

Bacteria genera such as *Legionella*, *Salmonella* and *Pseudomonas*, commonly causing cases and outbreaks in the tropics, but also in the rest of the world [16,17] were excluded. Τhe review endeavours to focus on infections transmitted by pool waters and caused by pathogens that, with the exception of *Cryptosporidium* spp. [18], are unusual in the moderate climates and common in the tropical and subtropical countries, including southern regions of Japan and North Australia, as shown in Figure 1. 

Other eligibility criteria were: that the studies were published in English, though we did allow a few notable exceptions to this rule; and that the publications in question were scientific papers and reviews in scientific journals, national and international public health platforms, and journals and platforms related to tourism (for example UNWTO). PubMed, Google Scholar, Science Direct, CDC, ECDC and WHO platform and publications were systematically searched.

Further to the aforesaid eligibility criteria, we reviewed 83 studies on cases and outbreaks in tropical countries, 45 studies on modes and trends of pathogen transmission and selected outbreaks in western countries, and 3 studies on trends in the tourist industry. In addition, information was harvested from official national and international websites. They are presented in groups according to the pathogen involved. As mentioned above, viruses transmitted by vectors that breed in waters were also considered as waterborne pathogens.

## 3. Results

### 3.1. Assessments of Swimming Pools in Developing Tropical Countries

The repeated reference to health problems deriving from the use of swimming pools could be related to sub-optimal regulations, which do not address all factors contributing to the swimmers’ well-being in a particular geographical area, or are poorly applied. For instance, countries around the Mediterranean basin are among the most popular tourist destinations. In a study, investigating pool and spa regulations in these countries, the conclusions were that the Africa and Middle East countries of this region possess satisfactory regulations comparable to the regulations of the European countries in the same area [19]. Similar conclusions were drawn from a report commissioned by Clúster de la Indústria Química de les Illes Balears in which a few countries worldwide were picked at random and their regulations presented and compared. Some tropical countries seem to adapt their regulations to specific issues. For instance, in the Mexican regulation, free living amoebae are included in the standard as water quality indicators along with bacterial parameters [20]. According to the authors of both reviews, the major question was whether regulations were applied, and if controls of the water quality and hygiene in the facilities were carried out by authorities.

Information on the monitoring and the assessment of swimming pool waters in countries of the tropical and subtropical zones is limited (Table 1). It is possible that monitoring is carried out in some countries, but scarcely few data have been published. From the North Africa countries some studies have been published from Egypt [21,22,23,24] starting in the 1960s. In the Middle East, certain subtropical countries such as Israel, Palestine and Jordan provided some limited monitoring data [25,26,27] including a study from Palestine on the presence of fungi in pool water and facilities [28]. Setsoafia Saba et al. published a water quality assessment of swimming pools and the risk of spreading infections in Ghana, which is one of the very rare publications in Sub-Saharan Africa [29]. From the Asian countries, China [30], and most often Iran [31,32], have published assessments which include bacterial indicators, some tropical parasites and fungi related to the sanitary quality in the facilities [31,32]. In light of the above, the aim of the present review is to update our knowledge of waterborne outbreaks in the tropical and subtropical zones of the main tropical pathogens transmitted through the use of swimming pools, spas and hot tubs with a particular emphasis on tourist facilities.

### 3.2. Microsporidia

Microsporidia are newly emerging pathogens of humans and animals. They are tiny obligate intracellular parasitic fungi and as such are often still managed by diagnostic parasitology laboratories. Due to the small size of their spores and uncharacteristic staining properties they are difficult to detect. Accordingly, epidemiological studies to elucidate the sources of human-pathogenic Microsporidia and their routes of transmission are difficult to perform [33]. Faecal-oral transmission is the likely route of infection in humans with intestinal microsporidiosis [34]. The last two decades have seen several publications related to ocular microsporidiosis, in particular those forms affecting the cornea. Both immunocompetent, immunocompromised and AIDS patients are vulnerable to the acquisition of microsporidia and especially to keratoconjunctivitis, which is usually seen in immunocompromised individuals or in contact lens wearers. The organism is widespread in the environment and is considered a waterborne pathogen [34,35]. Exposure to soil, muddy water, and minor trauma are possible risk factors. 

An analysis of risk factors for microsporidiosis showed that swimming in pools comprises an additional significant risk factor [36], even though conventional levels of chlorine (1–3 mg/L) used in swimming pools where water temperatures normally reach or exceed 22 °C should be adequate to greatly reduce or eliminate the infectivity *of* microsporidial species *E. intestinalis*, *E. hellem* and *E. cuniculi* spores after relatively short exposure times [37]. In Paris (France), in a survey of pools for microsporidia, *Cryptosporidium* spp. and *Giardia* spp., microsporidia were detected in only one out of 48 water samples [38]. 

The tropics seem to host most of the cases of microsporidial keratitis. A high prevalence has been documented in Singapore [39] and in India [40] and transmission through contact with water has been suggested. The present review identified 2 published studies (Table 2) clearly relating infection to the presence of microsporidia in pools in the tropics. In Paris, Curry et al. referred to a case of an HIV-negative patient from Bangladesh with bilateral keratitis who was found to be infected with a microsporidial parasite belonging to the genus *Nosema*. The patient had bathed in a rural pond 7 days prior to the development of ocular symptoms. *Nosema* parasites are common insect parasites and the source of this microsporidial infection was possibly from mosquito larvae developing in the pond in which the patient bathed [41]. In Taipei, Taiwan, a retrospective study included 10 eyes of 9 immunocompetent patients diagnosed with microsporidial keratitis. All of them were known to contract this disease after bathing in hot springs. The nine patients travelled and bathed in at least four different spa resorts located in two different areas [42].

### 3.3. Parasites 

Waterborne parasitic protozoan diseases are distributed worldwide and comprise, in both developed and developing countries, reasons for epidemic and endemic human suffering. Looking at the trends of the prevalence of parasitic diseases in the developed world a significant decrease has been observed, which may be attributed to the substantial improvements in data reporting and the establishment of surveillance systems [43,44]. The highest prevalence of parasitic protozoan infections is known to occur in developing countries due to lower hygiene standards. In addition, developing countries that are more likely to be most affected by such waterborne disease outbreaks still lack reliable surveillance systems, and an international standardization of surveillance and reporting systems has yet to be established [45]. In 1999, the European Network on Imported Infectious Diseases Surveillance (TropNetEurop) was set up in order to collate reliable data on imported infectious diseases to Europe and assess trends over time [46].

A review by Lim et al. provided a comprehensive overview of the available data and studies on waterborne parasite occurrences among the Association of Southeast Asian Nations (ASEAN), which is comprised of ten member states (i.e., Brunei Darussalam, Cambodia, Indonesia, Lao People’s Democratic Republic (PDR), Malaysia, Myanmar, the Philippines, Singapore, Thailand, and Vietnam) with the aims of identifying ways in which to progress. Many of these countries are booming tourist destinations. Swimming pools are included as a source of transmission. He points out the fact that there are massive gaps of knowledge in the occurrence, morbidity and mortality associated with parasitic diseases [47]. According to a review providing data related to neglected parasitic protozoa in the tropics reporting that only an estimated 1% of global outbreaks of waterborne parasitic protozoa outbreaks have occurred in Asia, it is evident that there is a paucity of information from this region where organized mechanisms of documentation of parasitic infections or waterborne outbreaks are lacking [48]. *Cryptosporidium*, *Amoebae* and *Schistosoma* spp. are the parasites with the highest public health significance when swimming pools are the route of transmission.

#### 3.3.1. Schistosoma spp.

Schistosomiasis is caused by diagenetic blood trematodes. The three main species infecting humans are *Schistosoma haematobium*, *S. japonicum*, and *S. mansoni*. Two other species, more localized geographically, are *S. mekongi* and *S. intercalatum*. Οther species of schistosomes, which parasitize birds and mammals, can cause cercarial dermatitis in humans [49]. Acute schistosomiasis was first described in 1847 in the prefecture of Katayama, Hiroshima district, Japan. A woman brought to the region to be married was found to become acutely unwell with a fever after she had been exposed to fresh water. Snails in fresh waters contribute to the life cycle of *Schistosoma* as, under optimal conditions, the eggs hatch and release miracidia, which swim and penetrate specific snail intermediate hosts [49].

Schistosomiasis has been rare in Europe and there is very limited published literature dealing with relevant outbreaks. In Corsica one outbreak involving 120 people infected after swimming in a fresh water swimming pool is one of the rare published cases [50]. Nevertheless, schistosomiasis is increasingly imported into temperate climates by immigrants and travellers to endemic areas [51,52,53,54]. Schistosomiasis in returning travellers is one of the most common imported tropical infections with potentially serious complications, which are preventable upon early diagnosis [55]. Human contact with water is required for infection by schistosomes. 

Grobusch et al. studied imported schistosomiasis in Europe by seeking data from TropNetEurop. Three hundred and thirty-three reports of schistosomiasis have been analysed for their epidemiological and clinical features. The majority of patients were of European origin (53%), who travelled predominantly for tourism to endemic areas (52%). The majority of infections were acquired in Africa; 92 (%) infections were attributed to *Schistosoma haematobium* [56]. However, in a 15-year observational study at the Hospital for Tropical Diseases, London, the prevalence of schistosomiasis in presenting travellers is decreasing with predominant species *S. haematobium* [55].

Schistosomiasis is one of the endemic diseases that take advantage of environmental modifications due to water conveyance in the Saharan countries, for example Burgina Faso [57]. In Egypt, risk factors for *S. haematobium* infection were male gender, an age <21 years old, living in small communities, and exposure to canal water [58].

One of the first published reports on an epidemic of acute schistosomiasis concerned travellers returning from Mali. Imported schistosomiasis acquired in the Dogon country in Mali, West Africa, was first demonstrated in 1989 in three Spanish travellers [59]. More recently, 79 cases of acute schistosomiasis were reported by the Hospital of Tropical Diseases, London, between 1998 and 2012. Most of these cases were young, male travellers who acquired their infection in Lake Malawi (53%). Most of the other cases were from West Africa, with only 13% acquiring their disease in East Africa, one in North Africa (Libya), and two in the Middle East (Saudi Arabia, Yemen). Most were on holiday (68%), while 16% had been working as volunteers. All of them reported contact fresh water in an area where schistosomiasis is endemic [60].

The present review identified five published studies clearly regarding schistosomiasis transmitted via swimming pools in the tropics, of which three were related to tourism (Table 2). In 1993, a 35-year-old Belgian woman was admitted to the University Hospital of Antwerp with schistosomiasis symptoms. She had swum with a group of travellers in a water pool in the Dongon valley in Mali. Sixty-two per cent (eight people) of the 13 travellers had acquired Schistosoma infection; seven of them had developed Katayama syndrome. All travellers, except one, who acquired a *Schistomoma* infection, had swum for at least 5 min in the pool [61]. In a study from the area of Belo Horizonte, Brazil, a group of 18 individuals was included. They had the impression that the water was clean and no snails were observed. *S. mansoni* was transmitted from non-symptomatic positive residents through infected intermediate hosts to visitors. The visitors came from an urban area who had never had contact with the disease before and who developed acute schistosomiasis [62]. Also in Brazil, transmission occurred in a non-endemic area of Brazil, which became a new point of transmission due to the immigration of infected workers [63]. In Upper Benue Valley in Cameroon, swimming in a pool for the local population was significantly associated with schistosomiasis infection [64]. The Department of Infectious Diseases, University Hospital of Leiden, The Netherlands, reported an outbreak of schistosomiasis among non-immunized travellers. Of 30 travellers in two consecutive groups, 29 who had swum in freshwater pools in the Dogon area of Mali, West Africa, were monitored for 12 months. Twenty-eight (97%) of those became infected; 10 (36%) of the 28 had cercarial dermatitis, and in 15 (54%), Katayama fever developed [65].

#### 3.3.2. *Cryptosporidium spp.*

Transmission of *Cryptosporidium* has been on the increase over the last two decades. Currently, 31 valid *Cryptosporidium* species have been recognized and of these more than 17 have been found to infect humans. The most commonly reported species in humans worldwide are *C. parvum* and *C. hominis* [66]. This parasite has a low infectious dose, a small size that enables it to bypass water filtration systems, and resistance to chlorine disinfection at levels routinely used at swimming pools, water parks, and interactive fountains. It is the leading cause of outbreaks associated with disinfected recreational water and has also caused outbreaks in child care facilities. *Cryptosporidium* has the ability to cause community-wide outbreaks when transmitted in these venues [67] Swimming pool associated cases and outbreaks of cryptosporidiasis have been reported abundantly in the western world [68,69,70,71,72].

The burden of cryptosporidiosis is higher in tropical countries. In Australia, for instance, cryptosporidiosis seems to be an endemic problem in warm, remote areas and in Aboriginal and Torres Strait Islander population-dominated regions [73]. The most recent Global Burden of Disease Study listed *Cryptosporidium* as an important cause of disease and death of children under 5 years of age in Sub-Saharan Africa [74]. From 2004 to 2010, 199 outbreaks of human gastroenteritis due to the waterborne transmission of 59 enteric parasitic protozoa were reported worldwide and of these, *Cryptosporidium* spp. was the etiological agent in 60.3% of the outbreaks [60,61]. Bathing in contaminated swimming and therapeutic pools is a major mode of waterborne transmission of *Cryptosporidium* and other pathogens [75].

In a recently published review, Ryan et al. found that the necessary key barriers to limiting swimming-pool associated outbreaks of cryptosporidiosis (lack of uniform national and international standards, poor adherence and understanding of regulations governing staff and patron behaviour, and low levels of public knowledge and awareness) are not widely applied [76]. The present review identified three published studies clearly reporting cryptosporidiosis transmitted via swimming pools, or reporting detection of Cryptosporidium in pool waters, in tropical countries (Table 2).

A study of 35 pools in Beijing, including some hotel pools, showed that 16.7% and 15% were positive for *Cryptosoridium* oocysts and *Giardia* cysts, respectively [66]. Also, in the Philippines, in a total of 33 water samples taken from various environmental sources, including swimming pools, 45.5% were positive for *Cryptosporidium.* Two hundred seventy three children developed cryptosporidiosis after using a pool. Later on the same children used 10 swimming pools in a different prefecture and four of them were infected [77]. In Broom, Western Australia, another outbreak of cryptosporidiosis involving children who swam at the public pool was described [78].

#### 3.3.3. *Acanthamoeba*, *Naegleria Species*

Free-living amoebae belonging to the genera *Acanthamoeba*, *Balamuthia*, *Naegleria* and *Sappinia* are important causes of disease in humans and animals. *Naegleria fowleri* produces an acute, and usually lethal, central nervous system (CNS) disease called primary Amoebic meningoencephalitis. *Acanthamoeba* spp. are opportunistic free-living amoebae capable of causing granulomatous amoebic encephalitis (GAE) in individuals with compromised immune systems [79]. *Acanthamoeba* spp., the Trojan horse of the microbial world, as it carries viruses, has two stages in its life cycle, an active trophozoite stage that exhibits vegetative growth and a dormant cyst stage with minimal metabolic activity. It is a causative agent of cutaneous lesions and sinus infections, vision-threatening keratitis and a rare but fatal encephalitis, known as granulomatous amoebic encephalitis [80]. *Acanthamoebae* and *Naegleria fowleri* are commonly found in warm freshwater environments such as hot springs, lakes, natural mineral water, and resort spas frequented by tourists. In an early survey of 13 swimming pools in Belgium, *Acanthamoeba* strains were detected in 43.6% of the samples [81]. Similarly, amoebae were detected in 27/30 swimming pools in New York State [82]. Previously thought to be a rare condition, the number of reported Primary Amoebic meningoencephalitis cases is increasing each year [83].

The present review identified 13 published studies reporting detection of free-living amoebae in pool waters in tropical countries [77,84,85,86,87,88,89,90,91,92,93,94,95] (Table 2). The earlier survey appeared in 1983 [84] and reported the presence of pathogenic and free-living amoebae in swimming pool waters of Mexico City. Among the organisms isolated, in their cystic or in their trophic stage, were *Naegleria fowleri Carter* and *Acanthamoeba castellanii Douglas* [84]. One study in Taiwan reported a fatality caused after swimming in hot springs [85]. The most recent one was carried out in two swimming pools in Alexandria (Egypt) [86].

### 3.4. Leptospira spp.

Leptospirosis also belongs to the spectrum of travel-related infections. Leptospirosis is a bacterial zoonosis caused by host-dependent spirochetes of the genus *Leptospira*, which is widespread throughout the world. Its main sources are rodents, particularly rats, which excrete the spirochete *Leptospira* spp. in urine. Humans are infected by direct contact with urine of infected animals or by contact with an infected environment such as surface water [96].

Leptospirosis is an important re-emerging tropical disease, especially in areas with a notable military presence. Several epidemics of leptospirosis have been reported worldwide during the past century, while leptospirosis is endemic in most of the urban areas in Southern and Western India, where outbreaks usually occur after flooding caused by heavy seasonal rainfall [97]. Almost every country in South and Southeast Asia, South and Central America and several island nations across the world are endemic to leptospirosis [98]. It is endemic in Sub-Saharan Africa; however, for most countries scarce epidemiological data, if any, exist.

The disease has been increasingly reported in travellers, particularly those travelling to tropical areas, due to the development of fresh-water sports and leisure activities [99,100], Leptospirosis is often reported in travellers to South Africa [96] and in travellers from Sub Saharan Africa. Returning from a water sports holiday in South Africa, a 49-year old man presented with acute leptospirosis [101]. In a recently published review from Rajarata University, Sri Lanka, the authors pointed out that a clear increase in the proportion of travel-associated leptospirosis over the time was observed. According to their review, the countries with the highest number of cases detected in travellers returning from endemic regions are the US, Netherlands, Japan, France, Germany and Australia; among reports of systematically collected country level data, Israel reported the highest incidence of travel associated leptospirosis (41.7%) [102]. In a hospital study in Paris, France, fifteen cases of travel-related leptospirosis were reported. All travellers except one were returning from holidays in the tropics (seven from SE Asia, three from Sub-Saharan Africa, two from Reunion Island). The most frequent at-risk exposure was bathing in fresh water [103]. The clinical course of a leptospirosis outbreak at the Hash House Harriers Club on Guam, Micronesia, in the western Pacific Ocean, has been reported. Patients declared multiple exposures to wet river banks, mud, and swamps [104].

One case clearly connected a leptospirosis case to swimming in a pool (Table 2). A 25 year-old German woman visiting the Dominican Republic and staying for 3 weeks in a village became infected when swimming in the heavily chlorinated swimming pool during a trip to Samana [105].

### 3.5. Viruses

Viruses are considered a significant cause of recreationally associated waterborne diseases with a number of relevant outbreaks in western countries [106,107,108,109]. However, they have been difficult to document because of the wide variety of illnesses associated and limitations in detection methods. *Noroviruses* are the largest cause of outbreaks with just under half of the outbreaks occurring in swimming pools (49%) [110]. Some sporadic publications refer to transmission of *Hepatitis A virus (HAV)* [111,112] and *Echovirus* 30 [113]. 

The present review identified six published studies reporting the viruses’ detection in swimming pool waters in tropical countries [114,115,116,117,118,119] (Table 2). The earliest was a study of a primary school outbreak of pharyngoconjunctival fever attributed to swimming in the swimming pool of a school camp [114]. Swimming pool water contaminated with *Human Adenovirus serotype 4 (HAdV-4)* was the most likely source of infection, although one instance of likely person-to-person transmission was noted [119].

### 3.6. Indirect Role of Swimming Pools in Water Related Diseases

Vector-borne diseases are human illnesses caused by parasites, viruses and bacteria that are transmitted by mosquitoes, sandflies, triatomine bugs, blackflies, ticks, tsetse flies, mites, snails and lice, causing more than 700,000 deaths globally. All the major vector-borne diseases, together, account for around 17% of all infectious diseases [8]. Swimming pools are considered major contributors to the disease burden, as proliferation sites for mosquitoes like *Anopheles*, transferring Malaria; *Aedes*, transferring *Dengue*, *yellow fever* and the *Zika viruses*; *Culex* transferring West Nile Fever. The latter became a major problem after the first cases and epidemics in western countries, starting in New York in 1999 [120]. Concerning the ecology of the *Culex* mosquitoes, Petersen et al., in their review of the spread of *West Nile Virus*, included density of poorly maintained swimming pools in the critical factors for *Arvoviral* proliferation [121]. Chen et al., in their review of the significance of the *Zika virus* as a new public health concern, emphasize the need to eliminate standing waters outside homes, including swimming pools [122].

Distribution of vector-borne diseases is determined by complex demographic, environmental and social factors. Global travel and trade, unplanned urbanization and making the transmission season longer or more intense or causing diseases to emerge in countries where they were previously unknown [8]. The world risk maps, demonstrated that for dengue fever Sub-Saharan areas and the central and northern countries of South America are the most dangerous zones [123]. Mackenzie et al., in his risk map, presents the spread and resurgence of Japanese encephalitis, West Nile and dengue encompassing all continents except Antarctica [124]. An important outbreak of dengue fever occurred recently in France [125], and of the *West Nile virus* in Greece [126]. 

Vectors are contributing to the dispersion of lethal diseases in the tropics, initially in agricultural and rural areas [127,128]. There are reports of westerners traveling in Southeast Asian countries who were infected by the *Zika virus*—an Australian traveller to Indonesia [129], a Canadian traveller to Thailand [130]—and the trends of the disease alter as time goes by with the symptoms getting more severe [131]. Nevertheless, in a review by De Sylva and Marshall dealing with the factors contributing to urban malaria transmission in Sub-Saharan Africa, the authors concluded that “artificial rather than natural vector breeding sites provide the most abundant sources of mosquito larvae in African urban centres. Africa’s demography is rapidly changing with a fast increasing number of people moving to urban areas”. According to the same review, urban malaria is considered an emerging problem in Africa because the populations of most large African cities have grown exponentially over the last 30 years. Ninety-five artificial vector breeding sites are referred to in this review, including swimming pools, in contrast with only 42 natural sites [132].

The present review identified five published studies reporting surveys of swimming pools in the tropics as environments encouraging vector’s proliferation (Table 2). Impoinvil et al. conducted larval surveys in habitats located in urban Malindi, Kenya, and, out of the 250 habitats sampled, 66 were unused swimming pools. Of the 110 habitats found to be positive for mosquitoes, unused swimming pools represented 42.7% and 148 anopheline pupae were found in eight of the 66 unused swimming pools while none was found in the other habitats [133]. The same authors in an earlier study reported that, from a total of 889 *Anopheles* and 7217 culicine immatures found in diverse water body types, unused swimming pools comprised 61% of all water bodies found to serve as the main habitats for Anopheles immatures [134]. Studies have been carried out in Dakar [135] and in Brazil [136].

### 3.7. Chlamydia Trachomatis

Trachoma, caused by *Chlamydia trachomatis*, has been noted throughout history as a significant cause of blindness. *Chlamydia trachomatis* is one of four bacterial species in the genus and they are obligatory intracellular parasites. Trachoma is considered a neglected tropical disease. The causative organism is passed from person to person by flies, fomites and fingers, particularly among preschool-aged children [137]. In 1998, the World Health Assembly adopted the goal of Global Elimination of Trachoma as a cause of blindness; the year 2020 was set as the target date by a WHO Alliance set up to support the elimination agenda [138]. 

Interestingly, Warren et al. notes the suggestion that the construction of well-maintained and chlorinated public swimming pools could be a practical method to improve facial cleanliness and disinfection and consequently reduce trachoma rates among children in remote Indigenous communities in Australia [139]. Nevertheless, simultaneous use by travellers might enhance transmission of *Chlamydia trachomatis* through the use of the pool, even though such a case has not been reported to the present. As with all obligatory intracellular parasites, *C. trachomatis* is unlikely to be affected by chlorination and thus survives well in pool water. Ozone only, in a concentration of 4 ppm, was enough to inactivate *C. trachomatis* and *C. pneumonia* according to a study conducted mostly for medical purposes [140].

## 4. Discussion

Global experience and research demonstrate that international travel can pose various risks to health, depending both on the health needs of the traveller and on the type of travel undertaken. Travellers may encounter sudden and significant changes in altitude, humidity, temperature and exposure to a variety of infectious diseases, which can result in illness. In addition, serious health risks may arise in areas where accommodation is of poor quality, hygiene and sanitation are inadequate, medical services are not well developed and clean water is unavailable [2]. Trends in the West towards novel, alternative “natural” ponds, deprived of disinfection with chemicals and using instead biological processes for cleaning the organic compounds in the water are expanding rapidly. Many European countries have adopted guidelines or regulations specific for natural ponds. These establishments require careful, scientifically sound management [141]. Some or all of these factors apply in numerous tropical and subtropical countries of the developing world. Also, the trend towards higher standard accommodation in tourist establishments and more water-intense activities—including the use of pools and hot springs—coincides with changes in the global climate system leading to declining water resources and poorer water quality in many regions [142]. Tourists’ lack of awareness of their impact on the environment becomes an added factor aggravating sustainability in certain destinations [7]. 

A host of viral, bacterial, fungal, helminth and protozoal diseases that occur mainly in the tropics and subtropics remain neglected, and hence the phrase “neglected tropical diseases” is used to characterize them [9]. The growing number of people travelling to the tropics means that it is imperative that there be more effective management of various public health issues by local authorities in the countries concerned, as well as by international bodies, tourist agencies and social groups, to enhance prevention and protection. Travellers should be considered as an integral part of the global surveillance network for emerging infections. Research and the knowledge gained can be used to alert the global community to the presence or susceptibility patterns of pathogens in different regions; to inform strategies that can be used to control infections in developing countries; and to prepare travellers to those areas and guide the care for those returning [4]. 

Tropical diseases transmitted via swimming pools and hot spring waters are less well understood than the hazards deriving from the use of unsafe food or drinking water. Also, secondary transmission occurs when travellers return to their homeland. Today, transmission at home is frequently related to climate change. Any climate change may alter the disease burden resulting from exposure to pathogens transmitted through recreational waters. In a study examining the impact of temperature, humidity, and precipitation on the incidence of reported West Nile virus infections in the US, increasing weekly maximum temperature and weekly cumulative temperature were similarly and significantly associated with a higher incidence of reported WNV infections [143].

Eighty three studies dealing with *Microsporidia*, *Leptospira*, *Schistosoma*, *Cryptosporidium*, *Amoebae*, viruses, and vectors breeding in swimming pools and hot springs in the tropics, and fulfilling pre-set criteria, have been included in this survey of the literature. The survey indicated that papers dealing with the quality of pool waters in the tropics are scarce, and information about infected tourists using pools is even less. The published literature, at least in international languages, presenting assessments and surveys of swimming establishments in the developing world are also scarce. Reasons for this neglect seem to be manifold including, as noted above, lesser awareness on this issue, not only in the developing, but also in the developed world. On the other hand, it is true that cases and outbreaks have been difficult to document because by its very nature travelling makes it difficult to follow up the cases in question and to carry out an epidemiological investigation. An international collaboration on this issue, along the lines of the European Legionnaires’ Disease Surveillance Network (ELDSNet), would certainly be an important step forward. As emphasized in a review published by RIVM, it is becoming imperative that recreational waterborne infectious diseases be prioritized and quantified [144].

Castor and Beach have made several recommendations for the prevention and control of disease transmission in swimming venues. They recommend the redesign of aquatic facilities, increased governmental oversight of swimming pool maintenance and training of staff, and education of the public regarding healthy swimming habits. In addition, they recommend that high-risk groups, such as the elderly and infirm and pregnant women, should be made aware of their increased risk of illness as a result of swimming, even in apparently adequately disinfected swimming waters [145]. Tourists in general, and in particular tourists travelling to the tropical and subtropical countries of the developing world, should be included in the list of vulnerable citizens and provided with specific advice, services and care.

## 5. Conclusions

There is abundant information and guidance to travellers regarding precautions that need to be taken in respect of food, drinking water and air quality in tropical destinations. Nevertheless, information on the hazards presented by recreational and especially pool, spa and hot spring waters as a mode of transmission of pathogens is limited. The survey indicated that papers dealing with the quality of pool waters in the tropics are scarce, and information about infected tourists using pools is even less. In addition, the ongoing climate change may alter the disease burden resulting from exposure to pathogens transmitted through recreational waters. Under the pressure of a growing number of people undertaking international travel, and yet faster growth of such travel in the tropical and subtropical zones, assessments of the swimming pool establishments, research and prevention strategies for pool safety in the tropics are imperative. Public health authorities need to provide guidance to westerners travelling to exotic destinations on how to protect their health in swimming pools. An international collaboration on this issue would certainly be an important step forward.

## Figures and Tables

**Table 1:** Assessments of swimming pools (SPs) located in tropical and subtropical countries.

Location/Country	Positive Results	Ref.
North Africa, Egypt	The authors suggest artificial plastic SPs as a prophylactic measure against infection with schistosomiasis in developing countries.	[21]
North Africa, Assiut Town, Egypt	In a survey of 2 SPs, which included 50 water samples *Dermatophytes*, *Aspergyllus* sp., *Penicillium*, *Altenaria*, *Syncephalastrium*, *Mucor* were detected.	[22]
North Africa, Alexandria, Egypt	Assessment of the environmental and health aspects of some SPs. Presence of pathogens indicated.	[23]
North Africa, Alexandria, Egypt	Assessment of 5 SPs, 30 water samples. Compliance of pool water with regulations regarding bacterial indicators was 56.7%. In 10% of the samples *Cryptosporidium* oocysts and *Giardia* cysts were detected.	[24]
Middle East, Ein Feshka, Dead Sea, Israel	Medical report of 10 cases of *Mycobacterium marinum* mimicking leismaniasis. Most of the infections were contracted in natural bathing pools.	[25]
Middle East, West Bank, Palestine	An assessment of 58 water samples, collected from 46 SPs. All unacceptable according to regulations. 21/23 water samples were positive for *Salmonella* spp.	[26]
Middle East, Amman, Jordan	Assessment of 85 SPs in Amman. Compliance of the pools’ water with the microbial parameters was 56.5%.	[27]
Middle East, Nablus district, Palestine	In a survey of 3 SPs, 50 keratinophilic fungal species were recovered. The most frequently isolated species were *Acremonium strictum* & *Cladosporium cladiosporioides.* The most abundant species were *Acremonium strictum*, and *Aspergillus flavus.*	[28]
Sub Saharan Africa, Ghana	In a survey of 7 SPs, faecal coliforms, *E. coli*, total heterotrophic bacteria were recovered from all SPs; *E. coli O157:H7* were recovered from 2 SPs. Antibiotic resistance tests revealed the highest resistance was in sulfamethoxazole (46%).	[29]
Asia, Guangzhou, China	A survey of 39 municipal SPs revealed protozoa (12.8%), *P. aeruginosa* (69.2%), total coliforms, *E. coli* (4%), *Cryptosporidium* & *Giardia* (12.8%), *E. coli O157*, *Shigella*, and *Salmonella*.	[30]
Asia, Ahwaz Iran	In a survey of 10 indoor SPs, 593 water and environmental samples (shower areas, dressing rooms, pool walls, slippers) revealed 372 saprophytic fungi species and 32 yeasts. The most common were *Aspergillus* & *Penicillium*.	[31]
Asia, Shahrekord City, Iran	In a seasonal assessment of 2 indoor SPs (459 pool water, shower & dressing room samples) faecal coliform *Pseudomonas aeruginosa*, *Legionella*, *Escherichia coli* and Heterotrophic Plate Count values exceeded regulations. The most prevalent fungi were in the showers, the most frequent being *Aspergillus* spp. (48.91%).	[32]

**Table 2:** List of surveillance studies of swimming pools (SPs), and respective cases and outbreaks of infections associated with swimming pools and hot springs in tropical and subtropical countries.

***Microsporidia***			
**Location/Country**	**Type of Research**	**Positive Results**	**Reference**
Rural areas, Bangladesh	Report of an incident of a traveller from Bangladesh returning to Paris, France	A man suffered from bilateral keratitis after bathing in a rural pond. The patient was found to be infected with a microsporidial parasite belonging to the genus *Nosema.*	[41]
Taipei, Taiwan	Retrospective study of patients diagnosed with microsporidial keratitis	All patients were known to have contracted microsporidial keratitis after bathing in hot springs.	[42]
***Schistosoma*** **spp.**			
**Location/Country**	**Type of Research**	**Positive Results**	**Reference**
Dogon Valley, Mali	Study of an acute schistosomiasis in Belgian travellers returning from Dogon Valley, Mali	8/13 travellers infected with *Schistosoma*. 5/8 travellers had experienced swimmer’s itch and developed Katayama syndrome.	[55]
Belo Horizonte, State of Minas Gerais, Brazil	Study of an outbreak of acute schistosomiasis in a holiday resort at an endemic area	17 cases infected with *S. mansoni.*	[56]
São João del Rei, Brazil	Study of an outbreak where an area became infected due to influx of infected workers from endemic areas, who infected water sources, including SPs	50 workers infected in the pool with *S. mansoni.*	[57]
Upper Benue Valley, North Cameroon	Study of the risk factors for human schistosomiasis in the local population	High prevalence of the disease depending on, among other factors, the intensity of contact with the water.	[58]
Dogon Valley, Mali	Study of an outbreak in two groups of 30 Dutch travellers returning from Dogon area of Mali where they swam in fresh water pools	29 infected with *S. intercalatum*, *S. haematobium.*	[59]
***Cryptosporidium*** **spp.**			
**Location/Country**	**Type of Research**	**Positive Results**	**Reference**
Beijing, China	Survey of 35 randomly selected hotel SPs, 60 water samples	16.7% positive for *Cryptosporidium*, 15% positive for *Giardia.*	[66]
Various areas, Philippines	Survey of water sources including SPs	33% positive for *Cryptosporidium.*	[77]
Broome, Kimberley region, Western Australia	Investigation of outbreak of cryptosporidiasis	11/18 cases swam in the public pool. In faecal and pool water samples *Cryptosporidium ominis* was identified.	[78]
***Acanthamoebae*** ***& Naegleria Species***			
**Location/Country**	**Type of Research**	**Positive Results**	**Reference**
Mexico City, Mexico	Survey of six swimming pools	All SPs were positive for *Acanthamoebae*. The most commonly found were *Amoebae* of the species *Naegleria gruberi Schardinger*.	[84]
Taichung, Taiwan	Diagnosis of fatality	One fatal case of meningoencephalitis caused by *N. fowleri* and transmitted in hot springs was reported.	[85]
Alexandria, Egypt	Survey of two SPs	Both SPs were positive for *Acanthamoeba* spp. and *Naegleria* spp.	[86]
Kuala Lumpur, Malaysia	Survey of 14 pools. Four water samples and six samples using swabs were collected from each	*Acanthamoeba* species were detected in all sampling sites of all SPs, while *Naegleria* spp. was detected in 3 sampling sites of 8 SPs.	[87]
Mexico City, Mexico	Survey of three physiotherapy tubs and 11 SPs	All therapy tubs were positive for *Acanthamoeba* spp., while 7/11 SPs were positive for *Naegleria* spp.	[88]
Brazil, Porto Alegre	Survey of 65 water samples from SPs	*Amoebae* were detected in 20% of the SPs. 4/65 water samples were positive for *Acanthamoeba* spp. while 9/65 water samples were positive for free-living amoebae.	[89]
Egypt, various locations	Survey in various waters including two SPs	49.2% of pool water samples were positive for heat-tolerant *Acanthamoeba* spp.	[90]
Porto Alegre, Brazil	Survey in pools and spas	8/72 water samples were positive for *Acanthamoeba* spp. distributed in group genotypes T3, T5, T4, T15.	[91]
Brasilia District, Brazil	Study of the pathogenicity of strains from environmental sources	4/4 *Acanthamoeba* spp. isolates from pool waters were pathogenic.	[92]
Ahwaz, Iran	Survey of 110 water and soil samples including four SPs	In 71.6% of water samples *Acanthamoeba* spp. was detected SP isolates belong to T4 genotype.	[93]
Various areas, Philippines	Survey of rivers, ponds, dispensers, wells, taps, natural lakes and SPs	33.3% of SP water samples were positive for *Acanthamoeba* sp. While 9.1% of SP water samples were positive for *Naegleria* spp.	[77]
Adana, Afyon, Kutahya, Mersin and Nigde provinces, Turkey	Survey of hot springs and SPs	42% of water samples were positive for *Acanthamoeba* sp. belonging to T3, T4, T5 genotypes.	[94]
Malaysia Peninsular	A survey of recreational lakes, streams, SPs	*Naegleria sp* was detected in all samples.	[95]
***Leptospira*** **spp.**			
**Location/Country**	**Type of Research**	**Positive Results**	**Reference**
Various places, Dominican Republic	Study of leptospirosis in travellers	A German woman developed leptospirosis after swimming in a chlorinated SP.	[105]
***Viruses***			
**Location/Country**	**Type of Research**	**Positive Results**	**Reference**
Queensland, Australia	Study of a primary school outbreak of pharyngo-conjunctival fever attributed to swimming in the SP of a school camp	40% of the students infected by *Adenovirus type 3.*	[114]
Pretoria, South Africa	A study of the risk of infection of *HAdVs* detected in a survey of 3 SPs, 92 water samples	*HAdVs* were detected in 15 samples.	[115]
Porto Alegre, Brazil	Survey of SPs for the detection of adenoviruses in *Acanthamoeba* strains	16 *Acanthamoeba* strains were detected, *HAdVs* were detected in 62.5% (10/16) of *Acanthamoeba* isolates.	[116]
South Africa	Investigation of an outbreak related to swimming in the school camp pool	90 children & the SP water were positive for *Echovirus 3.*	[117]
Taiwan, various areas	A study to determine the prevalence of *HAdVs* in hot springs. 57 hot springs and 14 public SPs were investigated, 57 water samples	*HAdVs* were detected in 28.1% of the samples from hot springs and 21.4% of SP water samples.	[118]
Beijing, China	A study of an outbreak of pharyngoconjunctival fever related to swimming in a University SP	50 patients used the same SP. *HAdV* type 4 was identified from the patients and SP water samples.	[119]
***Vectors***			
**Location/Country**	**Type of Research**	**Positive Results**	**Reference**
Malindi, Kenya	A systematic review of the factors contributing to urban transmission of malaria in Sub-Saharan Africa	*Anopheles gambiae* proliferating in SPs. Artificial rather than natural breeding sites provide most abundant sources for mosquito larvae.	[132]
Malindi, Kenya	A study on larvae surveys in urban environments and the productivity of unused SPs in relation to other habitats	Unused SPs accounted for 42.7% of all 110 positive habitats. *Anopheles gambiae s.l.* and *Culex quinquefasciatus* were detected.	[133]
Malindi, Kenya	A study on the abundance of immature *Anopheles* and culicines in various water body types in the urban environment	Unused SPs comprised 21.7% of water bodies serving as habitats for immature *Anopheles*.	[134]
Dakar, Senegal	An entomological survey on the determinants of malaria transmission in the city of Dakar	355 private properties were visited, including SPs. *Culicidae* larvae were found in 80 (23%) and *Anopheles* larvae in 11 (3%).	[135]
Sao Jose de Rio Preto, Brazil	A study on the evaluation of two sweeping methods for estimating the number of immature *Aedes aegypti*	*Aedes aegypti* was harvested in various types of containers including SPs.	[136]

## References

[1] (2017). World Tourism Organization, Tourism Highlights. http://www2.unwto.org/publication/unwto-tourism-highlights-2017.

[2] (2012). World Health Organization, International Travel and Health,. http://www.who.int/ith.

[3] World Health Organization (2006). Water, Sanitation and Health Team. Guidelines for Safe Recreational Water Environments. Volume 2, Swimming Pools and Similar Environments.

[4] Baker D.M. (2015). Tourism and the health effects of infectious diseases: Are there potential risks for tourists?. Int. J. Saf. Secur. Tour./Hosp..

[5] Barna Z., Kádár M. (2012). The risk of contracting infectious diseases in public swimming pools. A review. Annali dell Istituto Superiore di Sanità.

[6] TravelHealthPro. https://travelhealthpro.org.uk/.

[7] Page J.S., Essex S., Causevic S. (2014). Tourist Attitudes Towards Water Use in the Developing World: A Comparative Analysis. Tour. Manag. Perspect..

[8] World Health Organization: WHO. www.who.int.

[9] Utzinger J., Becker S.L., Knopp S., Blum J., Neumayr A.L., Keiser J., Hatz C.F. (2012). Neglected tropical diseases: Diagnosis, clinical management, treatment and control. Swiss Med. Wkly..

[10] Charrel R.N., de Lamballerie X., Raoult D. (2007). Chikungunya outbreaks—The globalization of vector borne diseases. N. Engl. J. Med..

[11] Rammaert B., Beauté J., Borand L., Hem S., Buchy P., Goyet S., Guillard B. (2011). Pulmonary melioidosis in Cambodia: A prospective study. BMC Infect. Dis..

[12] Goodman R.A., Buehler J.W. (2009). Delinquent Mortgages, Neglected Swimming Pools and West Nile Virus, California. Emerg. Infect. Dis..

[13] Marten G., Harrison C., Nguyen M., Sacket S., Thompson G., Carroll M., Riegel C. (2013). The Use of Gambusia to Control Mosquito Larvae in Abandoned Swimming Pools: The New Orleans Experience. New Orleans Mosquito, Termite & Rodent Control Board. http://www.gerrymarten.com/publicatons/pdfs/GM_new-orleans-swimming-pools.pdf.

[14] Horney J., Goldberg D., Hammond T., Stone K., Smitherman S. (2017). Assessing the Prevalence of Risk Factors for Neglected Tropical Diseases in Brazos County, Texas. PLoS Curr..

[15] Caillouët K.A., Carlson J.C., Wesson D., Jordan F. (2008). Colonization of abandoned swimming pools by larval mosquitoes and their predators following Hurricane Katrina. J. Vector Ecol..

[16] Hlavsa M.C., Hill R.V., Beach J.M. (2016). Immediate closures and violations identified during routine inspections of Public Aquatic Facilities—Network for Aquatic Facility Inspection Surveillance, United States, 2013. MMWR Surveill. Summ..

[17] Hlavsa C.M., Cikesh L.B., Roberts A.V., Kahler M.A., Marissa M., Hilborn D.E., Wade J.T., Roellig M.D., Murphy L.J., Xiao L. (2018). Outbreaks Associated with Treated Recreational Water—United States, 2000–2014. MMWR Surveill. Summ..

[18] Chalmers R.M. (2012). Waterborne ourbreaks of cryptosporodiasis. Ann. Ist Super Sanita..

[19] Mavridou A., Pappa O., Papatzitze O., Blougoura A., Drossos P. (2014). An overview of pool and spa regulations in Mediterranean countries with a focus on the tourist industry. J. Water Health.

[20] CliQ: Towards a Clean Future. Swimming Pool Regulations and Pool Market Analysis in the Tourism Sector. https://cliqib.org.

[21] Abd-Rabbo H. (1968). A new suggestion. Artificial plastic swimming pools as prophylactic measures against infection with schistosomiasis in developing countries. J. Trop. Med. Hyg..

[22] Maghazy S.M.N., Abdel-Mallek A.Y., Bagy M.M.K. (1989). Fungi in Two Swimming Pools in Assiut Town, Egypt. Zentralbl. Mikrobiol..

[23] Abdou M.H., Akel M.M., El-Shal W.I., El-Naggar A.S. (2005). Study of the environmental health aspects of swimming pools in Alexandria City. J. Egypt. Public Health Assoc..

[24] Abd El-Salam M.M. (2012). Assessment of water quality of some swimming pools: A case study in Alexandria, Egypt. Environ. Monit. Assess..

[25] Even-Paz Z., Haas H., Sacks T., Rosenmann E. (1976). Mycobacterium marinum skin infections mimicking cutaneous leishmaniasis. Br. J. Dermatol..

[26] Al-Khatib I.A., Salah S. (2003). Bacteriological and chemical quality of swimming pools water in developing countries: A case study in the West Bank of Palestine. Int. J. Environ. Health Res..

[27] Rabi A., Khader Y., Alkafajei A., Aqoulah A.A. (2008). Sanitary conditions of public swimming pools in Amman, Jordan. Int. J. Environ. Res. Public Health.

[28] Ali-Shtayeh M.S., Khaleel T.K., Jamous R.M. (2003). Ecology of dermatophytes and other keratinophilic fungi in swimming pools and polluted and unpolluted streams. Mycopathologia.

[29] Courage Kosi Setsoafia Saba, Saviour Kojo Tekpor (2015). Water Quality Assessment of Swimming Pools and Risk of Spreading Infections in Ghana. Res. J. Microbiol..

[30] Wei X., Li J., Hou S., Xu C., Zhang H., Atwill E.R., Li X., Yang Z., Chen S. (2018). Assessment of Microbiological Safety of Water in Public Swimming Pools in Guangzhou, China. Int. J. Environ. Res. Public Health.

[31] Rafiei A., Amirrajab N. (2010). Fungal Contamination of Indoor Public Swimming Pools, Ahwaz, South-west of Iran. Iran. J. Public Health.

[32] Fadaei A., Amiri M. (2014). Comparison of Chemical, Biological and Physical Quality Assessment of Indoor Swimming Pools in Shahrekord City, Iran in 2013. Glob. J. Health Sci..

[33] Rinder H. (2004). Transmission of microsporidia to humans: Water-borne, food-borne, air-borne, zoonotic, or anthroponotic?. Southeast Asian J. Trop. Med. Public Health.

[34] Joseph J., Vemuganti G.K., Sharma S. (2005). Microsporidia: Emerging ocular pathogens. Indian J. Med. Microbiol..

[35] Dowd S.E., Gerba C.P., Pepper I.L. (1998). Confirmation of the human-pathogenic microsporidia Enterocytozoon bieneusi, Encephalitozoon intestinalis, and Vittaforma corneae in water. Appl. Environ. Microbiol..

[36] Hutin Y.J., Sombardier M.N., Liguory O., Sarfati C., Derouin F., Modaï J., Molina J.M. (1998). Risk factors for intestinal microsporidiosis in patients with human immunodeficiency virus infection: A case-control study. J. Infect. Dis..

[37] Li X., Fayer R. (2006). Infectivity of microsporidian spores exposed to temperature extremes and chemical disinfectants. J. Eukaryot. Microbiol..

[38] Fournier S., Dubrou S., Liguory O., Gaussin F., Santillana-Hayat M., Sarfati C., Molina J.M., Derouin F. (2002). Detection of Microsporidia, cryptosporidia and Giardia in swimming pools: A one-year prospective study. FEMS Immunol. Med. Microbiol..

[39] Sharma S., Das S., Joseph J., Vemuganti G.K., Murthy S. (2011). Microsporidial keratitis: Need for increased awareness. Surv. Ophthalmol..

[40] Vemuganti G.K., Garg P., Sharma S., Joseph J., Gopinathan U., Singh S. (2005). Is microsporidial keratitis an emerging cause of stromal keratitis? A case series study. BMC Ophthalmol..

[41] Curry A., Mudhar H.S., Dewan S., Canning E.U., Wagner B.E. (2007). A case of bilateral microsporidial keratitis from Bangladesh--infection by an insect parasite from the genus Nosema. J. Med. Microbiol..

[42] Fan N.-W., Wu C.-C., Chen T.-L., Yu W.-K., Chen C.-P., Lee S.-M., Lin P.-Y. (2012). Microsporidial Keratitis in Patients with Hot Springs Exposure. J. Clin. Microbiol..

[43] Karanis P., Kourenti C., Smith H. (2007). Waterborne transmission of protozoan parasites: A worldwide review of outbreaks and lessons learnt. J. Water Health.

[44] Baldursson S., Karanis P. (2011). Waterborne transmission of protozoan parasites: Review of worldwide outbreaks—An update 2004–2010. Water Res..

[45] Efstratiou A., Ongerth E.J., Karanis P. (2017). Waterborne transmission of protozoan parasites: Review of worldwide outbreaks—An update 2011–2016. Water Res..

[46] TropNet: European Network for Tropical Medicine and Travel Health. http://www.tropnet.net/.

[47] Lim A.L.Y., Nissapatorn V. (2017). Transmission of waterborne parasites in the Association of Southeast Asian Nations (ASEAN): Overview and direction forward. Food Waterborne Parasitol..

[48] Plutzer J., Karanis P. (2016). Neglected waterborne parasitic protozoa and their detection in water. Water Res..

[49] Centers for Disease Control and Prevention (CDC). https://search.cdc.gov/search/?query=schistosomes&sitelimit=&utf8=✓&affiliate=cdc-main.

[50] Boissier J., Grech-Angelini S., Webster B.L., Allienne J.F., Huyse T., Mas-Coma S., Toulza E., Barré-Cardi H., Rollinson D., Kincaid-Smith J. (2016). Outbreak of urogenital schistosomiasis in Corsica (France): An epidemiological case study. Lancet Infect. Dis..

[51] Meltzer E., Artom G., Marva E., Assous M.V., Rahav G., Schwartzt E. (2006). Schistosomiasis among travelers: New aspects of an old disease. Emerg. Infect. Dis..

[52] Clerinx J., Bottieau E., Wichmann D., Tannich E., Van Esbroeck M. (2011). Acute schistosomiasis in a cluster of travelers from Rwanda: Diagnostic contribution of schistosome DNA detection in serum compared to parasitology and serology. J. Travel. Med..

[53] Marchese V., Beltrame A., Angheben A., Monteiro G.B., Giorli G., Perandin F., Buonfrate D., Bisoffi Z. (2018). Schistosomiasis in immigrants, refugees and travellers in an Italian referral centre for tropical diseases. Infect. Dis. Poverty.

[54] Röser D., Bjerrum S., Helleberg M., Nielsen H.V., David K.P., Thybo S., Stensvold C.R. (2018). Adventure tourism and schistosomiasis: Serology and clinical findings in a group of Danish students after white-water rafting in Uganda. JMM Case Rep..

[55] Coltart C.E., Chew A., Storrar N., Armstrong M., Suff N., Morris L., Chiodini P.L., Whitty C.J. (2015). Schistosomiasis presenting in travellers: A 15 year observational study at the Hospital for Tropical Diseases, London. Trans. R. Soc. Trop. Med. Hyg..

[56] Grobusch M.P., Mühlberger N., Jelinek T., Bisoffi Z., Corachán M., Harms G., Matteelli A., Fry G., Hatz C., Gjørup I. (2003). Imported schistosomiasis in Europe: Sentinel surveillance data from TropNetEurop. J. Travel Med..

[57] Kpoda N.W., Sorgho H., Poda J.N., Ouédraogo J.B., Kabré G.B. (2013). Schistosomiasis caused by Schistosoma mansoni in the Kou valley: Characterization of the transmission system and socioeconomic impact. C. R. Biol..

[58] El-Khoby T., Galal N., Fenwick A., Barakat R., El-Hawey A., Nooman Z., Habib M., Abdel-Wahab F., Gabr N.S., Hammam H.M. (2000). The epidemiology of schistosomiasis in Egypt: Summary findings in nine governorates. Am. J. Trop. Med. Hyg..

[59] Corachan M., Ruiz L., Valls M.E., Gascon J. (1992). Schistosomiasis and the Dogon country (Mali). Am. J. Trop. Med. Hyg..

[60] Logan S., Armstrong M., Moore E., Nebbia G., Jarvis J., Suvari M., Bligh J., Chiodini P.L., Brown M., Doherty T. (2013). Acute Schistosomiasis in Travelers:14 Years’ Experience at the Hospital for Tropical Diseases, London. Am. J. Trop. Med. Hyg..

[61] Colebunders R., Verstraeten T., Van Gompel A., Van den Ende J., De Roo A., Polderman A., Visser L. (1995). Acute Schistosomiasis in Travelers Returning from Mali. J. Travel Med..

[62] Enk J.M., Amorim A., Schall T.V. (2003). Acute schistosomiasis outbreak in the metropolitan area of Belo Horizonte, Minas Gerais: Alert about the risk of unnoticed transmission increased by growing rural tourism. Mem. Inst. Oswaldo Cruz..

[63] Lambertucci J.R., Drummond S.C., Voieta I., de Queiróz L.C., Pereira P.P., Chaves B.A., Botelho P.P., Prata P.H., Otoni A., Vilela J.F. (2013). An outbreak of acute Schistosoma mansoni Schistosomiasis in a nonendemic area of Brazil: A report on 50 cases, including 5 with severe clinical manifestations. Clin. Infect. Dis..

[64] Ndassa A., Mimpfoundi R., Gake B., Paul Martin M.V., Poste B. (2007). Risk factors for human schistosomiasis in the Upper Benue valley, in northern Cameroon. Ann. Trop. Med. Parasitol..

[65] Visser L.G., Polderman A.M., Stuiver P.C. (1995). Outbreak of schistosomiasis among travelers returning from Mali, West Africa. Clin. Infect. Dis..

[66] Xiao S., Yin P., Zhang Y., Hu S. (2017). Occurrence of Cryptosporidium and Giardia and the Relationship between Protozoa and Water Quality Indicators in Swimming Pools. Korean J. Parasitol..

[67] Centers for Disease Control and Prevention: Parasites-Cryptosporodium (also known as ‘Crypto’). https://www.cdc.gov/parasites/crypto/.

[68] Coetzee N., Edeghere O., Orendi J., Chalmers R., Morgan L. (2008). A swimming pool-associated outbreak of cryptosporidiosis in Staffordshire, England, October to December 2007. Euro Surveill..

[69] Insulander M., Lebbad M., Stenström T.A., Svenungsson B. (2005). An outbreak of cryptosporidiosis associated with exposure to swimming pool water. Scand. J. Infect. Dis..

[70] Shields J.M., Gleim E.R., Beach M.J. (2008). Prevalence of Cryptosporidium spp. and Giardia intestinalis in Swimming Pools, Atlanta, Georgia. Emerg. Infect. Dis..

[71] Wheeler C., Vugia D.J., Thomas G., Beach M.J., Carnes S., Maier T., Gorman J., Xiao L., Arrowood M.J., Gilliss D. (2007). Outbreak of cryptosporidiosis at a California waterpark: Employee and patron roles and the long road towards prevention. Epidemiol. Infect..

[72] Ichinohe S., Fukushima T., Kishida K., Sanbe K., Saika S., Ogura M. (2005). Secondary Transmission of Cryptosporidiosis Associated with Swimming Pool Use. Jpn. J. Infect. Dis..

[73] Lal A., Cornish L.M., Fearnley E., Glass K., Kirk M. (2015). Cryptosporidiosis: A Disease of Tropical and Remote Areas in Australia. PLoS Negl. Trop. Dis..

[74] GBD Mortality Causes of Death Collaborators (2015). Global, regional, and national age-sex specific all-cause and cause-specific mortality for 240 causes of death, 1990–2013: A systematic analysis for the Global Burden of Disease Study 2013. Lancet.

[75] Dale K., Kirk M., Sinclair M., Hall R., Leder K. (2010). Reported waterborne outbreaks of gastrointestinal disease in Australia are predominantly associated with recreational exposure. Aust. N. Z. J. Public Health.

[76] Ryan U., Lawler S., Reid S. (2017). Limiting swimming pool outbreaks of cryptosporidiosis—The roles of regulations, staff, patrons and research. J. Water Health.

[77] Onichandran S., Kumar T., Salibay C.C., Dungca Z.J., Tabo A.H., Tabo N., Tan T.C., Lim A.Y., Sawangjaroen N., Phiriyasamith S. (2014). Waterborne parasites: A current status from the Philippines. Parasit. Vectors.

[78] Ng-Hublin J.S., Hargrave D., Combs B., Ryan U. (2015). Investigation of a swimming pool-associated cryptosporidiosis outbreak in the Kimberley region of Western Australia. Epidemiol. Infect..

[79] Centers for Disease Control and Prevention: Parasites-Acantamobea. https://www.cdc.gov/parasites/acanthamoeba/.

[80] Visvesvara G.S., Moura H., Schuster F.L. (2007). Pathogenic and opportunistic free-living amoebae: Acanthamoeba spp., Balamuthia mandrillaris, Naegleria fowleri, and Sappinia diploidea. FEMS Immunol. Med. Microbiol..

[81] De Jonckheere J.F. (1979). Pathogenic free-living amoebae in swimming pools: Survey in Belgium. Ann. Microbiol..

[82] Lyons T.B., Kapur R. (1977). Limax Amoebae in Public Swimming Pools of Albany, Schenectady, and Rensselaer Counties, New York: Their Concentration, Correlations, and Significance. Appl. Environ. Microbiol..

[83] Heggie T.W. (2010). Swimming with death: Naegleria fowleri infections in recreational waters. Travel. Med. Infect. Dis..

[84] Rivera F., Ramírez P., Vilaclara G., Robles E., Medina F. (1983). A survey of pathogenic and free-living amoebae inhabiting swimming pool water in Mexico City. Environ. Res..

[85] Su M.Y., Lee M.S., Shyu L.Y., Lin W.C., Hsiao P.C., Wang C.P., Lai S.C. (2013). A Fatal Case of Naegleria fowleri Meningoencephalitis in Taiwan. Korean J. Parasitol..

[86] Al-Herrawy A.Z., Khalil M.I., El-Sherif S.S., Omar F.A.E., Lotfy W.M. (2017). Surveillance and Molecular Identification of Acanthamoeba and Naegleria Species in Two Swimming Pools in Alexandria University. Egypt. Iran. J. Parasitol..

[87] Init I., Lau Y.L., Arin Fadzlun A., Foead A.I., Neilson R.S., Nissapatorn V. (2010). Detection of free living amoebae, Acanthamoeba and Naegleria, in swimming pools, Malaysia. Trop. Biomed..

[88] Rivera F., Ramírez E., Bonilla P., Calderón A., Gallegos E., Rodríguez S., Ortiz R., Zaldívar B., Ramírez P., Durán A. (1993). Pathogenic and Free-living Amoebae Isolated from Swimming Pools and Physiotherapy Tubs in Mexico. Environ. Res..

[89] Caumo K., Frasson A.P., Pens C.J., Panatieri L.F., Frazzon A.P., Rott M.B. (2009). Potentially pathogenic Acanthamoeba in swimming pools: A survey in the southern Brazilian city of Porto Alegre. Ann. Trop. Med. Parasitol..

[90] Al-Herrawy A., Bahgat M., Mohammed A., Ashour A., Hikal W. (2013). Morpho-Physiological and Biochemical Criteria of Acanthamoeba spp. Isolated from the Egyptian Aquatic Environment. Iran. J. Parasitol..

[91] Fabres L.F., Rosa Dos Santos S.P., Benitez L.B., Rott M.B. (2016). Isolation and identification of Acanthamoeba spp. from thermal swimming pools and spas in Southern Brazil. Acta Parasitol..

[92] Alves Dde S., Moraes A.S., Nitz N., de Oliveira M.G., Hecht M.M., Gurgel-Gonçalves R., Cuba C.A. (2012). Occurrence and characterization of Acanthamoeba similar to genotypes T4, T5, and T2/T6 isolated from environmental sources in Brasília, Federal District, Brazil. Exp. Parasitol..

[93] Rahdar M., Niyyati M., Salehi M., Feghhi M., Makvandi M., Pourmehdi M., Farnia S. (2012). Isolation and genotyping of acanthamoeba strains from environmental sources in Ahvaz city, Khuzestan province, southern Iran. Iran. J. Parasitol..

[94] Evyapan G., Koltas I.S., Eroglu F. (2015). Genotyping of Acanthamoeba T15: The environmental strain in Turkey. Trans. R. Soc. Trop. Med. Hyg..

[95] Ithoi I., Ahmad A.F., Nissapatorn V., Lau Y.L., Mahmud R., Mak J.W. (2011). Detection of Naegleria species in environmental samples from peninsular Malaysia. PLoS ONE.

[96] de Vries S.G., Visser B.J., Nagel I.M., Goris M.G., Hartskeerl R.A., Grobusch M.P. (2014). Leptospirosis in Sub-Saharan Africa: A systematic review. Int. J. Infect. Dis..

[97] Patil D.Y., Dahake R.V., Chowdhary A.S., Deshmukh R.A. (2017). Clinico-epidemiological observations of human leptospirosis from Mumbai, India. J. Infect. Public Health.

[98] Sehgal S.C. (2006). Epidemiological patterns of leptospirosis. Indian J. Med. Microbiol..

[99] Gelman S.S., Gundlapalli A.V., Hale D., Croft A., Hindiyeh M., Carroll K.C. (2006). Spotting the spirochete: Rapid diagnosis of leptospirosis in two returned travelers. J. Travel Med..

[100] Leshem E., Segal G., Barnea A., Yitzhaki S., Ostfeld I., Pitlik S., Schwartz E. (2010). Travel-Related Leptospirosis in Israel: A Nationwide Study. Am. J. Trop. Med. Hyg..

[101] Pimenta D., Democratis J. (2013). Risky behavior: A rare complication of an uncommon disease in a returning traveler. BMJ Case Rep..

[102] Bandara M., Ananda M., Wickramage K., Berger E., Agampodi S. (2014). Globalization of leptospirosis through travel and migration. Glob. Health.

[103] van de Werve C., Perignon A., Jauréguiberry S., Bricaire F., Bourhy P., Caumes E. (2013). Travel-related leptospirosis: A series of 15 imported cases. J. Travel Med..

[104] Brinker A.J., Blazes D.L. (2017). An outbreak of Leptospirosis among United States military personnel in Guam. Trop. Dis. Travel Med. Vaccines.

[105] Grobusch M.P., Bollmann R., Schönberg A., Slevogt H., Garcia V., Teichmann D., Jelinek T., Flick H., Bergmann F., Rosseau S. (2003). Leptospirosis in Travelers Returning from the Dominican Republic. J. Travel Med..

[106] Hauri A.M., Schimmelpfennig M., Walter-Domes M., Letz A., Diedrich S., Lopez-Pila J., Schreier E. (2005). An outbreak of viral meningitis associated with a public swimming pond. Epidemiol. Infect..

[107] D’Angelo L.J., Hieholzer J.C., Keenlyside R.A., Anderson L.J., Martone W.J. (1979). Pharyngoconjunctival fever caused by adenovirus type 4: Report of a swimming pool-related outbreak with recovery of virus from pool water. J. Infect. Dis..

[108] Maunula L., Kalso S., Von Bonsdorff C.H., Pönkä A. (2004). Wading pool water contaminated with both noroviruses and astroviruses as the source of a gastroenteritis outbreak. Epidemiol. Infect..

[109] Morbidity and Mortality Weekly Report (MMWR) (2004). An Outbreak of Norovirus Gastroenteritis at a Swimming Club—Vermont. Wkly Rep..

[110] Sinclair R.G., Jones E.L., Gerba C.P. (2009). Viruses in recreational water-borne disease outbreaks: A review. J. Appl. Microbiol..

[111] Mahoney F.J., Farley T.A., Kelso K.Y., Wilson S.A., Horan J.M., McFarland L.M. (1992). An outbreak of hepatitis A associated with swimming in a public pool. J. Infect. Dis..

[112] Tallis G., Gregory J. (1997). An outbreak of hepatitis A associated with a spa pool. Commun. Dis. Intell..

[113] Faustini A., Fano V., Muscillo M., Zaniratti S., La Rosa G., Tribuzi L., Perucci C.A. (2006). An outbreak of aseptic meningitis due to echovirus 30 associated with attending school and swimming in pools. Int. J. Infect. Dis..

[114] Harley D., Harrower B., Lyon M., Dick A. (2001). A primary school outbreak of pharyngo-conjunctival fever caused by adenovirus type 3. Commun. Dis. Intell..

[115] Van Heerden J., Ehlers M.M., Grabow W.O. (2005). Detection and risk assessment of adenoviruses in swimming pool water. J. Appl. Microbiol..

[116] Staggemeier R., Arantes T., Caumo K.S., Rott M.B., Spilki F.R. (2016). Detection and quantification of human adenovirus genomes in Acanthamoeba isolated from swimming pools. An. Acad. Bras. Cienc..

[117] Yeats J., Smuts H., Serfontein C.J., Kannemeyer J. (2005). Investigation into a school enterovirus outbreak using PCR detection and serotype identification based on the 5’ non-coding region. Epidemiol. Infect..

[118] Shih Y.J., Tao C.W., Tsai H.C., Huang W.C., Huang T.Y., Chen J.S., Chiu Y.C., Hsu T.K., Hsu B.M. (2017). First detection of enteric adenoviruses genotype 41 in recreation spring areas of Taiwan. Environ. Sci. Pollut. Res. Int..

[119] Li J., Lu X., Sun Y., Lin C., Li F., Yang Y., Liang Z., Jia L., Chen L., Jiang B. (2018). A swimming pool-associated outbreak of pharyngoconjunctival fever caused by human adenovirus type 4 in Beijing, China. Int. J. Infect. Dis..

[120] Komar N. (2003). West Nile virus: Epidemiology and ecology in North America. Adv. Virus Res..

[121] Petersen L.R., Brault A.C., Nasci R.S. (2013). West Nile Virus: Review of the Literature. JAMA.

[122] Chen H.L., Tang R.B. (2016). Why Zika virus infection has become a public health concern?. Chin. Med. Assoc..

[123] Rogers D.J., Wilson A.J., Hay S.I., Graham A.J. (2006). The Global Distribution of Yellow Fever and Dengue. Adv. Parasitol..

[124] Mackenzie J.S., Gubler D.J., Petersen L.R. (2004). Emerging flaviviruses: The spread and resurgence of Japanese encephalitis, West Nile and dengue viruses. Nat. Med..

[125] Succo T., Noel H., Nikolay B., Maquart M., Cochet A., Leparc-Goffart I., Catelinois O., Salje H., Pelat C., de Crouy-Chanel P. (2018). Dengue sero-survey after a 2-month long outbreak in Nîmes, France, 2015: Was there more than met the eye?. Euro Surveill..

[126] Patsoula E., Vakali A., Balatsos G., Pervanidou D., Beleri S., Tegos N., Baka A., Spanakos G., Georgakopoulou T., Tserkezou P. (2016). West Nile Virus Circulation in Mosquitoes in Greece (2010–2013). Biomed. Res. Int..

[127] Coulibaly B., Kone R., Barry M.B., Emerson B., Coulibaly M.B., Niare O., Beavogui A.H., Traore S.F., Vernick K.D., Riehle M.M. (2016). Malaria vector populations across ecological zones in Guinea Conakry and Mali, West Africa. Malar. J..

[128] Matthys B., Koudou B.G., N’Goran E.K., Vounatsou P., Gosoniu L., Koné M., Gissé G., Utzinger J. (2010). Spatial dispersion and characterization of mosquito breeding habitats in urban vegetable-production areas of Abidjan, Côte d’Ivoire. Ann. Trop. Med. Parasitol..

[129] Kwong J.C., Druce J.D., Leder K. (2013). Zika virus infection acquired during brief travel to Indonesia. Am. J. Trop. Med. Hyg..

[130] Fonseca K., Meatherall B., Zarra D., Drebot M., MacDonald J., Pabbaraju K., Wong S., Webster P., Lindsay R., Tellier R. (2014). First case of Zika virus infection in a returning Canadian traveler. Am. J. Trop. Med. Hyg..

[131] Salehuddin A.R., Haslan H., Mamikutty N., Zaidun N.H., Azmi M.F., Senin M.M., Syed Ahmad Fuad S.B., Thent Z.C. (2017). Zika virus infection and its emerging trends in Southeast Asia. Asian Pac. J. Trop. Med..

[132] De Silva M.P., Marshall J.M. (2012). Factors Contributing to Urban Malaria Transmission in Sub-Saharan Africa: A Systematic Review. J. Trop. Med..

[133] Impoinvil D.E., Mbogo C.M., Keating J., Beier J.C. (2009). The role of unused swimming pools as a habitat for Anopheles immature stages in urban Malindi, Kenya. J. Am. Mosq. Control Assoc..

[134] Impoinvil D.E., Keating J., Mbogo C.M., Potts M.D., Chowdhury R.R., Beier J.C. (2008). Abundance of immature Anopheles and culicines (Diptera: Culicidae) in different water body types in the urban environment of Malindi, Kenya. J. Vector Ecol..

[135] Gadiaga L., Machault V., Pagès F., Gaye A., Jarjaval F., Godefroy L., Cissé B., Lacaux J.P., Sokhna C., Trape J.F. (2011). Conditions of malaria transmission in Dakar from 2007 to 2010. Malar. J..

[136] Dibo M.R., Fávaro E.A., Parra M.C., Santos T.C., Cassiano J.H., Deitz K.V., Pagliotto A.M., Zini N., Benetti D.R., Chiaravalloti-Neto F. (2013). Evaluation of two sweeping methods for estimating the number of immature Aedes aegypti (Diptera: Culicidae) in large containers. Rev. Soc. Bras. Med. Trop..

[137] Courtright P., Rotondo L., MacArthur C., Jones I., Weaver A., Negash B.K., Olobio N., Binnawi K., Bush S., Abdala M. (2018). Strengthening the links between mapping, planning and global engagement for disease elimination: Lessons learnt from trachoma. Br. J. Ophthalmol..

[138] (1988). 51st World Health Assembly, Geneva, Resolution WHA51.11, Global Elimination of Blinding Trachoma,. http://www.who.int/blindness/causes/WHA51.11/en/.

[139] Warren J.M., Birrell A.L. (2016). Trachoma in remote Indigenous Australia: A review and public health perspective. Aust. N. Z. J. Public Health.

[140] Yamazaki T., Inoue M., Ogawa M., Shiga S., Kishimoto T., Hagiwara T., Matsumoto T., Hayashi T. (2004). Inactivation of Chlamydia trachomatis and Chlamydia (Chlamydophila) pneumoniae by ozone. Lett. Appl. Microbiol..

[141] Giampaoli S., Garrec N., Donzi G., Valeriani F., Erdinger L., Romano Spica V. (2004). Regulations concerning natural swimming ponds in Europe: Considerations on public health issues. J. Water Health.

[142] Gössling S., Peeters P., Hall M.C., Ceron J.-P., Dubois G., Lehmann V., Scot D. (2012). Progress in Tourism Management Tourism and water use: Supply, demand and security. An international review. Tour. Manag..

[143] Soverow J.E., Wellenius G.A., Fisman D.N., Mittleman M.A. (2009). Infectious Disease in a Warming World: How Weather Influenced West Nile Virus in the United States (2001–2005). Environ. Health Perspect..

[144] de Roda Husman A.M., Schets F.M. (2010). Climate Change and Recreational Water-Related Infectious Diseases, RIVM Publications,. http://hdl.handle.net/10029/258107.

[145] Castor M.L., Beach M.J. (2004). Reducing illness transmission from disinfected recreational water venues: Swimming, diarrhea and the emergence of a new public health concern. Pediatr. Infect. Dis. J..

